# Analysis of wideband tympanometry in Ménière’s disease

**DOI:** 10.1016/j.bjorl.2020.05.029

**Published:** 2020-07-21

**Authors:** Gisela Andrea Yamashita Tanno, Mônica Alcantara de Oliveira Santos, Marcelo Tabosa Dutra Sanches, Alessandra Spada Durante, Kátia de Almeida, Marcella Scigliano Gameiro, Nayara Michelle Costa de Freitas Roque, Osmar Mesquita de Sousa Neto

**Affiliations:** a Faculdade de Ciências Médicas Santa Casa de São Paulo (FCMSCSP), Departamento de Otorrinolaringologia, São Paulo, SP, Brazil; b Faculdade de Ciências Médicas Santa Casa de São Paulo (FCMSCSP), Curso de Fonoaudiologia, São Paulo, SP, Brazil

**Keywords:** Meniere’s disease, Tympanometry, Auditory tests, Acoustic impedance tests

## Abstract

**Introduction:**

Endolymphatic hydrops is the pathophysiological substrate of Ménière’s disease. The changes in the inner ear, transmitted to the middle ear through changes in the ossicular chain mobility, can be quantified by wideband tympanometry, through the measurement of the acoustic absorbance at multiple frequencies, represented by the sound energy absorbed by the middle ear, even at its early stages. Studying the behavior of the middle ear through the absorbance in patients with endolymphatic hydrops under ambient pressure and under peak pressure can be useful for detecting Ménière's disease.

**Objective:**

To characterize acoustic absorbance behavior in subjects with symptomatic and asymptomatic Ménière's disease compared to controls, in order to verify the ability of wideband tympanometry to detect Ménière's disease.

**Methods:**

We carried out a cross-sectional study with a diagnostic approach comparing the findings of wideband tympanometry at ambient pressure and peak pressure between the ears of the control group (n = 30), the asymptomatic group (n = 21) and the symptomatic group (n = 9).

**Results:**

Different peak pressure values were found between the ears of the control group (0 daPa), the asymptomatic group (−11 daPa) and the symptomatic group (−192 daPa), with *p* < 0.05 by the Kruskal-Wallis test, Mann Whitney test and Bonferroni correction. Different absorbance values were found between the ears of the symptomatic group and the asymptomatic group compared to the control group for low frequencies at ambient pressure and peak pressure, with *p* < 0.05 by the Kruskal-Wallis test, Mann Whitney test and Bonferroni correction.

**Conclusions:**

The Wideband Tympanometry test was capable of identifying the presence of Ménière´s disease, and to differentiate between asymptomatic and symptomatic patients, when comparing them with healthy individuals.

## Introduction

The acoustic immittance measurement is a valuable electroacoustic instrument in the detection of middle ear alterations, due to its speed and objectivity. It is characterized by the analysis of mechanical responses of the auditory system in response to acoustic stimulation and is related to the transfer of acoustic energy that occurs when sound waves reach the external auditory canal, when sound pressure is applied to the tympanic membrane, causing its movement. This measurement refers to the ease of or opposition to this flow of sound energy inside the auditory system.^1^

Wideband tympanometry emerged as a promise for a new way to assess middle ear conditions, being a simple, objective and non-invasive method. The frequency variations have an increased sensitivity for detecting small changes in the middle ear's acoustic mechanism, since high frequencies allow changes in the ossicular eardrum system to be evaluated.^1^

In Ménière's disease, changes in the ossicular chain mobility are expressed as a substrate of the increased pressure in the membranous labyrinth, which would restrict the mobility of the cochlear windows.^2^

Endolymphatic hydrops is the histopathological substrate of Ménière's disease, which is related to abnormalities in the production and absorption of fluids (endolymph and perilymph) and ions in the inner ear, characterized by the distention of the endolymphatic space.^3^

The diagnosis of Ménière's disease is eminently clinical. It is characterized by recurrent and spontaneous episodes of vertigo, fluctuating sensorineural hearing loss, tinnitus and aural fullness. Diagnostic assurance is only possible through *post-mortem* study of the temporal bone.^4^

Among the several measures that can be analyzed by wideband tympanometry is the acoustic absorbance, which consists of the ratio between the energy absorbed by the middle ear and the incident energy, presented in the external auditory canal. The absorbance consists of a real number between "zero" and "one", where "one" represents all the energy absorbed for the middle ear and can be expressed as a percentage.^5^

New equipment has appeared on the market with technologies capable of evaluating frequencies between 226 and 8000 Hz in three-dimensional (3D) mode, such as the Interacoustics Titan® equipment, called WideBand Tympanometry (WBT), which aims to perform a single recording able to obtain several forms of information, such as absorbance, tympanometric peak pressure and conventional tympanogram.

The aim of this study is to characterize the behavior of acoustic absorbance, in the wide ranges of frequency and pressure variation, in individuals diagnosed with symptomatic and asymptomatic Ménière's disease, compared with controls without Ménière's disease, aiming to verify the capacity of wideband tympanometry to detect clinical variations related to possible endolymphatic hydrops.

## Sample and methods

The present study project was analyzed and approved by the Research Ethics Committee, under Research protocol N. 1,704,766.

This research was a cross-sectional study, with a diagnostic focus, carried out from August 2016 to August 2017.

Inclusion criteria:

1) Patients with a definite clinical diagnosis of Ménière's disease, according to the criteria of the Bárány Society^6^: two or more episodes of spontaneous vertigo lasting between 20 min and 12 h; low and medium frequency sensorineural hearing loss documented by audiometry, defining the affected ear on at least one occasion: before, during or after a vertigo episode; fluctuating auditory signs (hearing loss, tinnitus or fullness) in the affected ear; and absence of another vestibular diagnosis that better explains the symptoms.
2) Patients with a probable clinical diagnosis of Ménière's disease, according to the criteria of the Bárány Society^6^: two or more episodes of vertigo or dizziness, lasting between 20 min and 24 h; fluctuating auditory symptoms (hearing loss, tinnitus or aural fullness) in the affected ear; absence of another vestibular diagnosis that can better explain the symptoms.
3) Patients with asymptomatic Ménière's disease on the day of the examination.
4) Patients with symptomatic Ménière's disease on the day of the examination, that is, with vertigo-type dizziness, associated with aural fullness and fluctuating hearing loss at the time of assessment.

Exclusion criteria:

1) Patients with neurological impairment;
2) Previous otologic surgeries;
3) Other otologic diseases.
4) Patients with mixed or conductive loss audiometry.

The sample consisted of 60 ears, 30 ears from the Control Group (CG) and 30 ears from the Study Group (SG) diagnosed with Ménière's disease, according to the criteria of the Bárány Society. Of these 30 ears in the study group, they were further subdivided into: Asymptomatic study group (AG), consisting of 21 ears, and Symptomatic study group (SYG), consisting of 9 ears.

**Figure 1:**
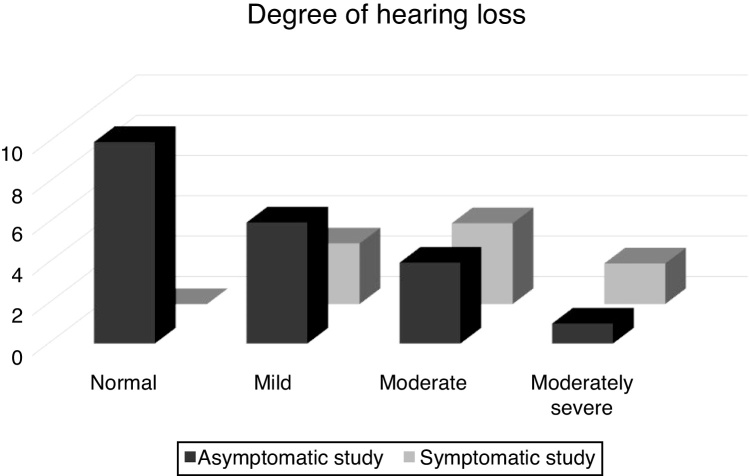
Distribution of the asymptomatic and symptomatic study groups in relation to the degree of hearing loss.

**Figure 2:**
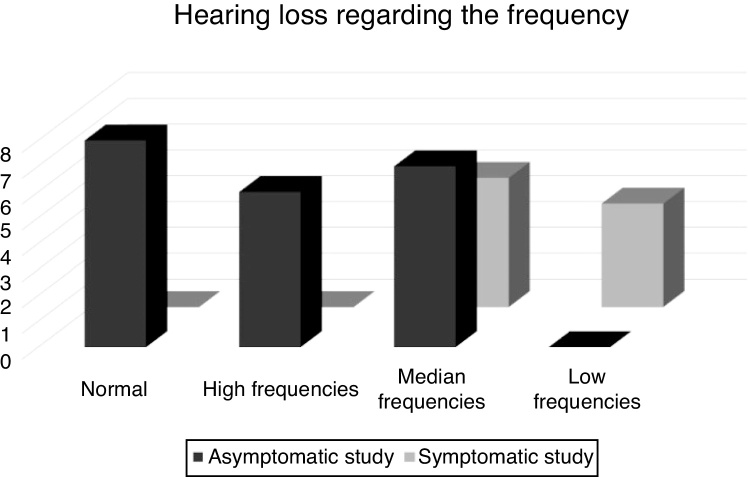
Distribution of asymptomatic and symptomatic study groups for hearing loss regarding the frequency.

When studying the association of hearing loss by comparing the Symptomatic Study Group (SYG) and the Asymptomatic Study Group (AG), it was observed that, in the SYG group, the predominance of sensorineural hearing loss was moderate and that, in the AG group, the auditory evaluation showed limits within the normal range and mild hearing loss, mostly, as shown in Fig. 1. When the hearing loss was found, it was verified, as shown in Fig. 2, that the sensorineural hearing loss at the low frequencies was present only in the SYG group and sensorineural hearing loss at high frequencies was present only in the AG group.

The ears in the control group consisted of 15 individuals without symptoms of dizziness or tinnitus, hearing loss, or a feeling of aural fullness in either ear. The ears of the study group were from 22 individuals.

In the control group, 13 individuals were females and 2 were males. In the study group, 18 individuals were females and 4 were males.

In the control group the individuals were aged from 21 to 54 years, with a median of 30 years, and a mean age of 33 years. In the study group, the individuals age ranged from 20 and 80 years, with a median of 54 years and a mean of 52 years.

To select the individuals, the researchers evaluated the patients at the Otoneurology Outpatient Clinic, including those who had a definite or probable clinical diagnosis of Ménière's disease according to the criteria of the Bárány Society, and invited patients who were already being followed to undergo the examination.

The individuals invited to participate in this study signed the Free and Informed Consent form, after they were clarified on the objectives and conditions involved in the study.

During the anamnesis, patients who denied a history of other ear diseases, without previous ear surgeries and neurological impairment underwent physical examination and otoscopy. The patients whose external auditory canal showed no alterations, an intact, translucent tympanic membrane, without bulging or retraction, without secretion in the middle ear, were referred to the Speech Therapy Clinic, where the researchers verified which patients were symptomatic or asymptomatic at the time of the examination, and performed wideband tympanometry in those who met the inclusion criteria in the sample using the Interacoustics Titan® equipment.

**Figure 3:**
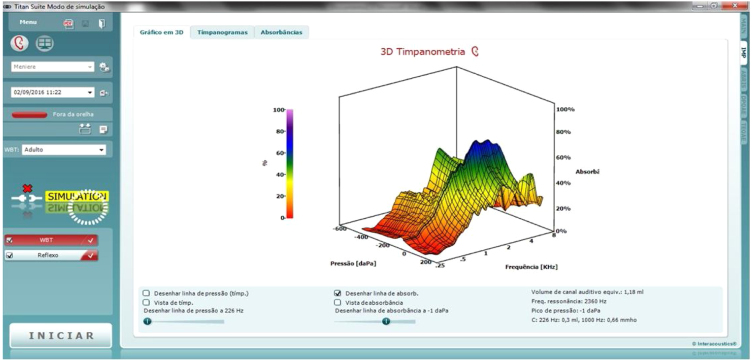
Example of 3D tympanometry (image extracted from the researchers' database).

**Figure 4:**
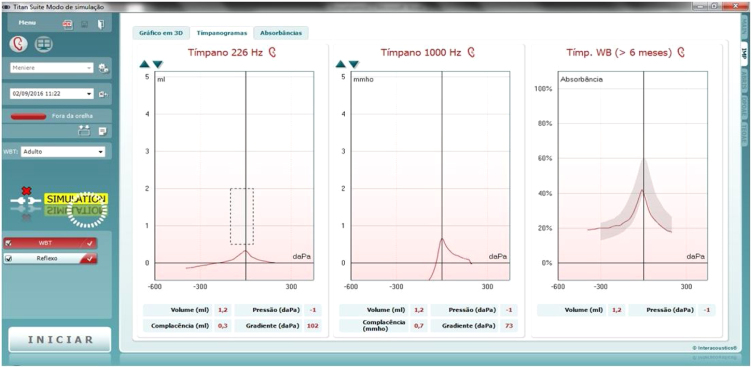
Example of traditional 2D tympanometry (image extracted from the researchers' database).

**Figure 5:**
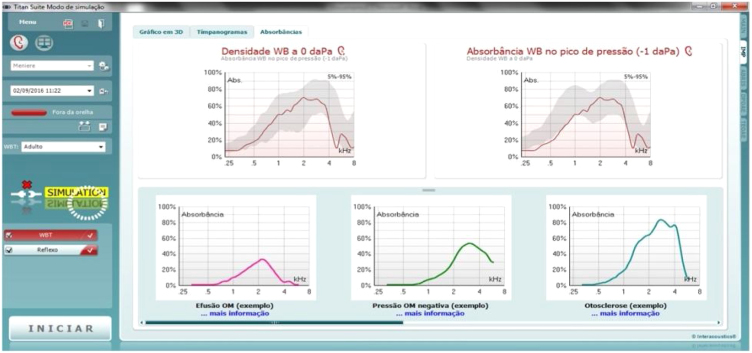
Example of absorbance (image extracted from the researchers' database).

In this study, we used the IMP440 module and the Clinical version, which performs tympanometry, ipsi- and contralateral reflexes, reflex decay, reflex latency, three Eustachian tube function tests, and has the optional wideband 3D and Excel modules for research, also allowing selecting the options for 3D visualization (Fig. 3), traditional 2D tympanometry (Fig. 4) or absorbance (Fig. 5).

The absorbance curve can be viewed over a wide frequency range, from 226 Hz to 8000 Hz.

To analyze the absorbance data at ambient pressure and at the peak, 17 frequencies were selected, of the 107 provided by the equipment, based on the studies of Shahnaz and Bork^7^ and Aithal et al.^8^ The frequencies of 226 Hz, 257 Hz, 324 Hz, 408 Hz, 500 Hz, 630 Hz, 794 Hz, 1000 Hz, 1260 Hz, 1587 Hz, 2000 Hz, 2520 Hz, 3175 Hz, 4000 Hz, 5040 Hz, 6350 Hz and 8000 Hz were selected.

## Results

The results were submitted to descriptive and inferential statistical analysis to characterize the recorded acoustic absorbance measurements and check whether there was a difference between the ears in the control group and the study group, asymptomatic and symptomatic, in the results of wideband tympanometry at ambient pressure and at peak pressure. The Mann-Whitney test, Kruskal-Wallis test and the Bonferroni correction were used for the inferential analysis of the results.

The Mann-Whitney and Kruskal-Wallis tests are non-parametric tests, used to compare two or more independent samples of equal or different sizes, of asymmetric distribution, with non-homogeneous variances. Bonferroni correction is used to decrease type I error, when comparing several groups. Type I error consists in rejecting the null hypothesis when it is true in a hypothesis test, that is, type I error is made when a result that has statistical significance is reached when, in fact, it happened by chance.^9^

The level of significance adopted for the study was 5% (*p* =  0.05).

### Comparison of pressure at the tympanometric peak between the CG, AG and SYG groups

**Figure 6:**
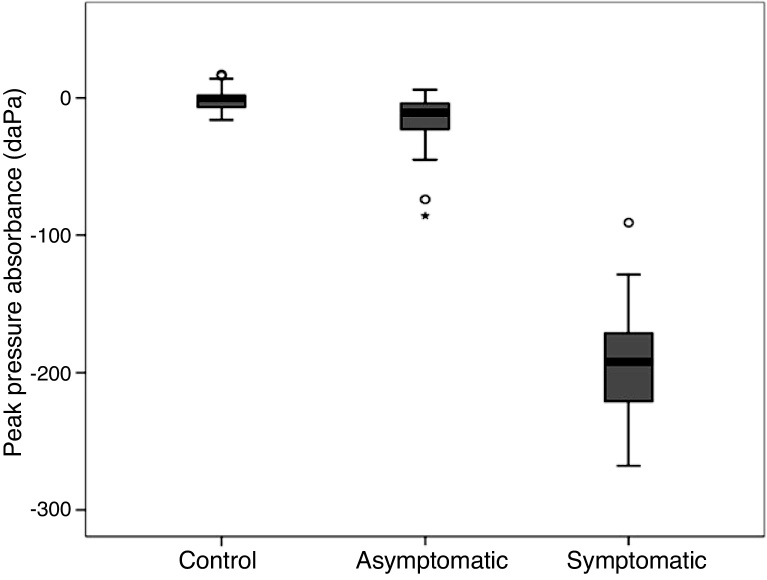
Box plot of peak pressure per group.

When studying the pressure at the peak of absorbance for each ear, the value of peak pressure in relation to the groups: Control (CG), Asymptomatic study (AG) and Symptomatic study (SYG), using the Kruskal-Wallis test, a difference was observed between peak pressure values in the CG, AG and SYG groups (*p* < 0.05) (Table 1 and Fig. 6).

**Table 1:** Comparison of peak pressure values in the ears according to the group.

Peak pressure (daPa)						
Group	Mean	Median	SD	Minimum	Maximum	*p*^a^
Control	−1.2	0.0	8.6	−16.0	17.0	<0.05
Asymptomatic	−19.0	−11.0	24.4	−86.0	6.0	
Symptomatic	−191.0	−192.0	55.7	−268.0	−91.0	

a Kruskal-Wallis test.

### Evaluation of absorbance values for the 17 frequencies at ambient pressure in the CG, AG and SYG groups

The absorbance values ​​were different between the CG, AG and SYG groups for the frequencies of 226 Hz, 257 Hz, 324 Hz, 408 Hz, 500 Hz, 630 Hz, 794 Hz, 1000 Hz, 1260 Hz, 2520 Hz, 3175 Hz and 4000 Hz, using the Kruskal-Wallis test. When performing the Bonferroni correction, it was observed that the absorbance values ​​were different between the CG, AG and SYG groups for the frequencies of 226 Hz, 257 Hz, 324 Hz, 408 Hz, 500 Hz, 630 Hz, 794 Hz and 1000 Hz, as shown in Table 2.

**Table 2:** Analysis of absorbance in the ears of the CG, AG and SYG groups, for the 17 frequencies at ambient pressure.

Absorbance					
Frequency	Control	Asymptomatic	Symptomatic	*p*^a^	*p*^b^
226 Hz	0.162	0.070	0.025	0.000001	0.000001
257 Hz	0.170	0.072	0.023	0.000001	0.000001
324 Hz	0.167	0.055	0.004	0.000001	0.000001
408 Hz	0.260	0.111	0.038	0.000001	0.000001
500 Hz	0.318	0.139	0.053	0.000001	0.000001
630 Hz	0.462	0.246	0.138	0.000001	0.000001
794 Hz	0.551	0.399	0.260	0.000001	0.000001
1000 Hz	0.699	0.569	0.448	0.000001	0.000001
1260 Hz	0.749	0.628	0.547	0.017	0.280
1587 Hz	0.642	0.633	0.631	0.667	1.000
2000 Hz	0.602	0.616	0.678	0.175	1.000
2520 Hz	0.595	0.643	0.884	0.020	0.340
3175 Hz	0.580	0.699	0.845	0.003	0.060
4000 Hz	0.493	0.584	0.800	0.005	0.080
5040 Hz	0.203	0.105	0.209	0.527	1.000
6350 Hz	0.318	0.181	0.239	0.071	1.000
8000 Hz	0.255	0.121	0.038	0.203	1.000

a Kruskal-Wallis test.

b Bonferroni correction.

**Figure 7:**
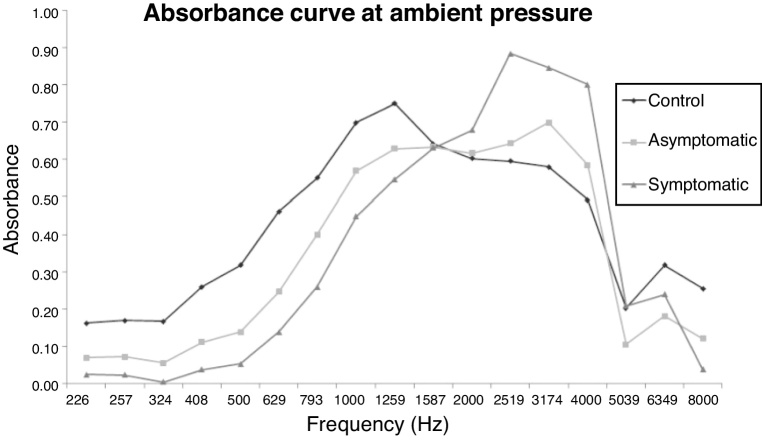
Comparison of the absorbance in the ears of the CG, AG, SYG groups, by frequency, at ambient pressure.

Table 3 and Fig. 7 showed the comparison of absorbance between the CG, AG and SYG groups by frequency at ambient pressure. When comparing the CG group and the AG, less absorbance was observed in the AG group compared to the CG group at the frequencies of 226 Hz, 257 Hz, 324 Hz, 408 Hz, 500 Hz, 630 Hz, 794 Hz, 1000 Hz and 1260 Hz, using the Mann-Whitney test and Bonferroni correction.

**Table 3:** Analysis of absorbance in the ears of the CG × AG, CG × SYG and AG × SYG groups, for the 17 frequencies at ambient pressure.

Absorbance						
Frequency	*p*^a^	*p*^b^	*p*^a^	*p*^b^	*p*^a^	*p*^b^
	CG × AG	CG × AG	CG × SYG	CG × SYG	AG × SYG	AG × SYG
226 Hz	0.000001	0.000001	0.000001	0.000001	0.001	0.236
257 Hz	0.000001	0.000001	0.000001	0.000001	0.002	0.266
324 Hz	0.000001	0.000001	0.000001	0.000001	0.003	0.266
408 Hz	0.000001	0.000001	0.000001	0.000001	0.014	0.334
500 Hz	0.000001	0.000001	0.000001	0.000001	0.027	0.465
630 Hz	0.000001	0.000001	0.000001	0.000001	0.028	0.462
794 Hz	0.000001	0.000001	0.000001	0.000001	0.044	0.416
1000 Hz	0.001	0.004	0.001	0.002	0.298	1.000
1260 Hz	0.010	0.046	0.053	0.085	0.483	1.000
1587 Hz	0.389	1.000	0.665	1.000	0.734	1.000
2000 Hz	0.811	1.000	0.152	0.306	0.039	0.211
2520 Hz	0.803	1.000	0.008	0.020	0.012	0.054
3175 Hz	0.051	0.161	0.002	0.004	0.081	0.269
4000 Hz	0.085	0.313	0.003	0.004	0.035	0.179
5040 Hz	0.267	0.824	0.987	1.000	0.497	1.000
6350 Hz	0.031	0.134	0.157	0.277	0.603	1.000
8000 Hz	0.257	0.736	0.094	0.298	0.493	1.000

a Mann-Whitney test.

b Bonferroni correction.

When comparing the CG group and the SYG, less absorbance was observed in the SYG group compared to the CG group at the frequencies of 226 Hz, 257 Hz, 324 Hz, 408 Hz, 500 Hz, 630 Hz, 794 Hz and 1000 Hz; higher absorbance was observed in the SYG group in relation to the CG group at the frequencies of 2520 Hz, 3175 Hz and 4000 Hz by the Mann-Whitney test and Bonferroni correction.

When comparing the AG and SYG groups, less absorbance was observed in the SYG group compared to the AG group at the frequencies of 226 Hz, 257 Hz, 324 Hz, 408 Hz, 500 Hz, 630 Hz, 794 Hz and greater absorbance was observed in the SYG group in comparison to the AG group at the frequencies of 2000 Hz, 2520 Hz and 4000 Hz by the Mann-Whitney test. When performing the Bonferroni correction, it was observed that there was no difference between the frequencies.

### Evaluation of the absorbance values for the 17 frequencies at peak pressure in the CG, AG and SYG groups

The absorbance values ​​were different in the CG, AG and SYG groups for the frequencies of 226 Hz, 257 Hz, 324 Hz, 408 Hz, 500 Hz, 630 Hz, 794 Hz and 1000 Hz by the Kruskal-Wallis test; when performing Bonferroni correction, it is observed that the absorbance values ​​were different in the three groups for the low frequencies of 226 Hz, 257 Hz, 324 Hz, 408 Hz, 500 Hz and 630 Hz, as shown in Table 4.

**Table 4:** Analysis of absorbance in the ears of the CG, AG, and SYG groups, for the 17 frequencies at peak pressure.

Absorbance					
Frequency	Control	Asymptomatic	Symptomatic	*p*^a^	*p*^b^
226 Hz	0.162	0.070	0.025	0.000001	0.000001
257 Hz	0.170	0.072	0.023	0.000001	0.000001
324 Hz	0.167	0.055	0.004	0.000001	0.000001
408 Hz	0.260	0.111	0.038	0.000001	0.010
500 Hz	0.318	0.139	0.053	0.000001	0.010
630 Hz	0.462	0.246	0.138	0.003	0.050
794 Hz	0.551	0.399	0.260	0.023	0.390
1000 Hz	0.699	0.569	0.448	0.033	0.570
1260 Hz	0.749	0.628	0.547	0.113	1.000
1587 Hz	0.642	0.633	0.631	0.417	1.000
2000 Hz	0.0602	0.616	0.678	0.366	1.000
2520 Hz	0.595	0.643	0.884	0.180	1.000
3175 Hz	0.580	0.699	0.845	0.171	1.000
4000 Hz	0.493	0.584	0.800	0.121	1.000
5040 Hz	0.203	0.105	0.209	0.466	1.000
6350 Hz	0.318	0.181	0.239	0.094	1.000
8000 Hz	0.255	0.121	0.038	0.570	1.000

a Kruskal-Wallis test.

b Bonferroni correction.

**Figure 8:**
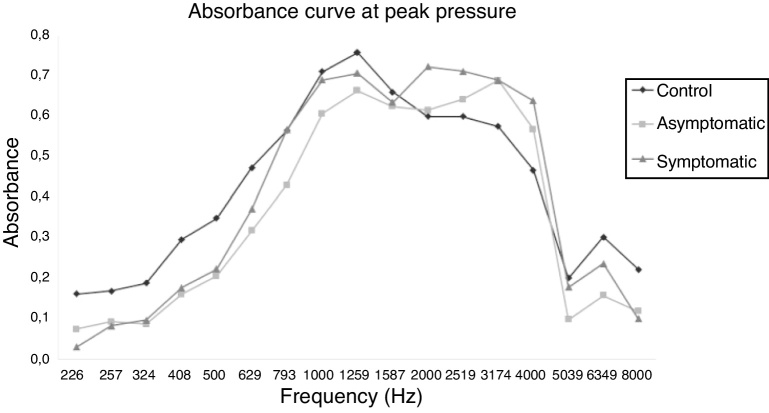
Comparison of the absorbance in the ears of the CG, SYG, CC groups, by frequency, at peak pressure.

Table 5 and Fig. 8 show the comparison of absorbance between the CG, AG and SYG groups, by frequency at peak pressure. When comparing the CG group and the AG, less absorbance was observed in the AG group compared to the CG group at the frequencies of 226 Hz, 257 Hz, 324 Hz, 408 Hz, 500 Hz, 630 Hz, 794 Hz, and 1000 Hz, whereas higher absorbance was observed in the SYG group in relation to the CG group at the mean frequency of 2520 Hz by the Mann-Whitney test. When performing the Bonferroni correction, less absorbance was observed in the AG group compared to the CG group at the frequencies of 226 Hz, 257 Hz and 324 Hz.

**Table 5:** Absorbance analysis in the ears of the CG × AG, CG × SYG and AG × SYG groups, for the 17 frequencies at peak pressure.

Absorbance						
Frequency	*p*^a^	*p*^b^	*p*^a^	*p*^b^	*p*^a^	*p*^b^
	CG × AG	CG × AG	CG × SYG	CG × SYG	AG × SYG	AG × SYG
226 Hz	0.000001	0.000001	0.000001	0.000001	0.039	0.448
257 Hz	0.000001	0.0003	0.000001	0.000001	0.526	1.000
324 Hz	0.001	0.004	0.000001	0.0001	0.946	1.000
408 Hz	0.007	0.052	0.000001	0.001	0.415	1.000
500 Hz	0.010	0.069	0.000001	0.0003	0.319	1.000
630 Hz	0.243	0.976	0.001	0.002	0.081	0.401
794 Hz	0.828	1.000	0.008	0.022	0.090	0.264
1000 Hz	0.677	1.000	0.012	0.031	0.103	0.387
1260 Hz	0.237	0.983	0.053	0.119	0.415	1.000
1587 Hz	0.764	1.000	0.203	0.569	0.428	1.000
2000 Hz	0.309	0.759	0.709	1.000	0.118	0.478
2520 Hz	0.039	0.205	0.909	1.000	0.230	0.361
3175 Hz	0.162	0.573	0.104	0.276	0.928	1.000
4000 Hz	0.067	0.157	0.193	0.637	0.277	1.000
5040 Hz	0.629	1.000	0.206	0.667	0.821	1.000
6350 Hz	0.264	0.559	0.031	0.121	0.946	1.000
8000 Hz	0.451	1.000	0.348	1.000	0.927	1.000

a Mann-Whitney test.

b Bonferroni correction.

When comparing the CG group and the SYG by the Mann-Whitney test, less absorbance was observed in the SYG group compared to the CG group at frequencies of 226 Hz, 257 Hz, 324 Hz, 408 Hz, 500 Hz, 630 Hz, 794 Hz, and 1000 Hz, whereas greater absorbance was observed in the SYG group compared to the CG group at the frequency of 6350 Hz. When performing the Bonferroni correction, lower absorbance was observed in the SYG group compared to the CG group at the low frequencies of 226 Hz, 257 Hz, 324 Hz, 408 Hz, 500 Hz, 630 Hz, 794 Hz, and 1000 Hz.

When comparing the AG and SYG groups using the Mann-Whitney test, less absorbance was observed in the SYG group compared to the AG group at 226 Hz. When performing the Bonferroni correction, it was observed that there was no difference between the frequencies.

## Discussion

### Absorbance peak pressure

When comparing peak pressure, the absorbance peak pressure measured by wideband tympanometry was evaluated. When comparing the peak pressure of the control group, the asymptomatic group and the symptomatic group, it was observed that the values ​​of the pressure at the peak increased markedly in the symptomatic group in relation to the asymptomatic and control groups; this could be the representation of the increased pressure of the endolymphatic system, caused by the histopathological changes found in endolymphatic hydrops.

Multifrequency tympanometry is the measurement of the impedance of the middle ear transmission system, with multiple frequencies from 200 Hz to 2000 Hz; it derives from individual vectors of the admittance complex (Y), which is the inverse of impedance, and its components (conductance and susceptibility) at different frequencies.^10^ Impedance (Z) in the middle ear system is a measure of resistance to the acoustic energy that flows from the ossicular chain to the inner ear. Admittance (Y) is the inverse of impedance and refers to the ease with which sound energy flows in an acoustic system and is a two-dimensional quantity represented by the vector sum of conductance (G) and susceptance (B). Conductance (G) represents resistance forces, while susceptance (B) represents reactive forces.^11^

When the middle ear point of resonance is found, mass and stiffness values ​​cancel each other out. The effect of stiffness in the system controls the transmission of low frequency sounds and the mass effect; the transmission of high frequency sounds, therefore, when evaluating the middle ear's acoustic immittance with the low frequency probe tone, which is mainly being investigated, is the result of the effect of the system stiffness on the passage of this sound; conversely, when using a high frequency tone, the assessed mechanism is the result of the system mass effect.^12^

Darrouzet et al. verified whether the measures of admittance (Y), susceptibility (B) and conductance (G) at 2 kHz could reflect the state of the annular ligament and cochlear pressure. They showed that the injection of a small amount of fluid in the tympanic scale of 22 guinea pigs increased cochlear pressure, leading to a peak division during the 2 kHz tympanometry, while the fluid aspiration caused the division peaks to disappear. When injecting again, the division peak reappeared and became more distinct and sharper, as the amount of injected fluid increased. Although the underlying mechanisms remain unknown, this experimental model demonstrated for the first time that variations in intracochlear pressure significantly modify the shape of tympanometry in a 2-kHz probe tones. A new method of analysis for measuring conductance or admittance with a 2-kHz tone probe, therefore, is suggested as being useful for the diagnosis of endolymphatic hydrops.^10^

Franco-Vidal et al. reported a potential use of multifrequency tympanometry as a new diagnostic test for the detection of endolymphatic hydrops. Forty patients with Ménière's disease outside of episodes and 24 individuals with normal hearing were evaluated. They found a significant difference between the affected ears of patients with Ménière's disease and normal ears, using the conductance width at 2000 Hz with a threshold of 235 daPa.^2^

Yasui et al. (2012) evaluated admittance with 2000-Hz probe tones in the diagnosis of ears with endolymphatic hydrops in patients with low frequency hearing loss. Thirty-six patients with low frequency hearing loss (including 21 with Ménière's disease, three with late endolymphatic hydrops and 12 with low frequency sensorineural hearing loss), 18 patients with other types of hearing loss and 16 individuals with normal hearing, were analyzed. They showed that the admittance width in the tympanogram at 2000 Hz in patients with low frequency hearing loss was significantly greater in ears with endolymphatic hydrops than in patients without it. Moreover, the widths in the ears with endolymphatic hydrops were greater than in the ears with other types of hearing loss. When the pressure was greater than 255 daPa, greater widths were observed in 38% of the ears with endolymphatic hydrops and in 21% of the ears with other types of hearing loss.^11^

Kato et al. investigated the association between the peak width in the tympanogram at 2000 Hz and the degree of endolymphatic hydrops on Magnetic Resonance Imaging (MRI) after intratympanic or intravenous gadolinium administration. The width of the peak conductance in ears with significant endolymphatic hydrops (observed by the MRI in the cochlea or in the vestibule, with pressure from 178.8 to 102.7 daPa) was greater than that observed in ears without endolymphatic hydrops. The peak width in ears with mild endolymphatic hydrops was not evident.^13^

In the present study, the peak absorbance pressure averaged −191 daPa, ranging from -268 to −91 daPa in the symptomatic group, thus demonstrating that the higher the pressure values, the more the tympanometric variables became evident.

### Absorbance at ambient pressure and peak pressures

When assessing the absorbance values ​​in relation to the frequencies, the ears were analyzed at ambient pressure and at the individual's ear tympanometric peak.

Comparing the absorbance in the control group, the symptomatic group and the asymptomatic group, a difference in absorbance was observed mainly at low frequencies; in the asymptomatic group and the symptomatic group, the absorbance was lower when compared to the control group. This reduction in absorbance in the asymptomatic and symptomatic groups can be understood as the representation of increased pressure in the inner ear, which, transmitted through the oval window to the ossicular chain, increased the stiffness of the ossicular tympanic system and reduced the conduction of low frequency stimuli.

In clinical practice, the probe tone most used in conventional tympanometry is 226 Hz. Generally, the pressure of +200 daPa (decaPascal) is applied to obtain the admittance values ​​in the middle ear because, at this pressure, the probe admittance is approximately equivalent to the air volume of the external auditory canal.^12^

The transmission of pressure variations from endolymph to perilymph has been poorly understood. The multifrequency tympanometry can measure perilymph pressure at high frequency (2000 Hz), which is in direct contact with the stapes platinum, of which impedance it can change. Consequently, there may be doubts about the capacity to analyze the endolymph pressure.

Kitahara et al. sought to clarify whether the low pressure chamber, used to treat patients with MD, could be used to diagnose the disease. Forty-five ears of patients with neurotologic disorders were studied. The individuals were placed in the supine position in a pressure chamber. The pressure was first decreased to -500 mm/H_2_O, and after 5 min, to −700 mm/H_2_O. The pressure was maintained at this level for 5 more minutes and then increased to 0 mm/H_2_O. This procedure was performed three times in a row. While the chamber pressure was below 0 mm/H_2_O, the subjects were instructed to abstain from actively balancing the middle ear pressure. When the chamber pressure was increased to 0 mm/H_2_O, they were instructed to balance the pressure of the middle ear and, if necessary, the Politzer maneuver or catheterization of the auditory tube was performed. As a result, hearing was improved in 50% of patients with Ménière’s disease after this procedure, demonstrating that variations in endolymphatic and perilymphatic pressure may be connected.^14^

The difference in absorbance was more accentuated when the data obtained at ambient pressure were analyzed in relation to the peak pressure, since the ambient pressure was the same for all, and did not change for the analyzed individuals, whereas the peak pressure varied according to the absorbance of each patient. Thus, the symptomatic group had higher peak pressure due to endolymphatic hydrops, and the absorbance curve in relation to the frequencies was similar to that of the other groups.

The fact that each patient has a different peak pressure can be associated with the variation in absorbance in relation to endolymphatic hydrops (*p* < 0.05), evident in a higher number of frequencies at ambient pressure than in the study with peak pressure. Therefore, the test can diagnose pressure changes in the inner ear, represented in the middle ear, due to lower absorbance, which impairs energy conduction by the ossicular system. These changes can be noticed at an early stage of these pressure variations, providing patients with earlier interventions.

Figure 7, Figure 8, which correlate the values of absorbance and frequency, showed these data more clearly, since the absorbance was lower in the symptomatic group compared to the symptomatic and control groups, and also in the asymptomatic group compared to the control group.

### Evaluation of the absorbance curve

When assessing the tracing of the absorbance curve in Figure 7, Figure 8, it was observed that the peak of absorbance of the individuals in the control group was between the frequencies of 1000 Hz, 1260 Hz, and 1587 Hz, while the peak of absorbance of the symptomatic group and asymptomatic group was between 2520 Hz and 3175 Hz. This was associated with the findings of endolymphatic hydrops because, the more accentuated the endolymphatic hydrops, the higher the pressure in the inner ear, the greater the stiffness in the cochlear windows, and the greater the stiffness in the ossicular tympanic system, which will be present at frequencies greater than 2000 Hz, with a consequent increase in absorbance at these frequencies. These alterations were more evident in symptomatic patients compared to patients in the asymptomatic and control groups, especially at ambient pressure, in which there was no pressure variation between patients, with peak absorbance between the frequencies of 2520 and 3175 Hz, as shown in Fig. 7 and, even at peak pressure, where there was an increased pressure value in patients with Ménière's disease, there was a formation of double peak absorbance at frequencies from 1000 Hz to 1587 Hz and at frequencies from 2520 to 3175 Hz, as shown in Fig. 8.

## Conclusion

The present study showed that wideband tympanometry is able to identify the presence of Ménière's disease and differentiate between asymptomatic and symptomatic patients, in comparison with individuals without the diagnosis of Ménière's disease.

## Conflicts of interest

The authors declare no conflicts of interest.
